# Influence of Sintering Temperature of Kaolin, Slag, and Fly Ash Geopolymers on the Microstructure, Phase Analysis, and Electrical Conductivity

**DOI:** 10.3390/ma14092213

**Published:** 2021-04-26

**Authors:** Nur Nadiah Izzati Zulkifli, Mohd Mustafa Al Bakri Abdullah, Anna Przybył, Paweł Pietrusiewicz, Mohd Arif Anuar Mohd Salleh, Ikmal Hakem Aziz, Dariusz Kwiatkowski, Marcin Gacek, Marek Gucwa, Jitrin Chaiprapa

**Affiliations:** 1 Faculty of Chemical Engineering Technology, University Malaysia Perlis (UniMAP), Perlis 01000, Malaysia; nadiahizzati.zulkifli@gmail.com (N.N.I.Z.); arifanuar@unimap.edu.my (M.A.A.M.S.); ikmalhakem@unimap.edu.my (I.H.A.); 2 Geopolymer & Green Technology, Center of Excellence (CEGeoGTech), University Malaysia Perlis (UniMAP), Perlis 01000, Malaysia; 3 Department of Physics, Częstochowa University of Technology, Dabrowskiego 69, 42-201 Częstochowa, Poland; anna.przybyl@pcz.pl (A.P.); pawel.pietrusiewicz@pcz.pl (P.P.); marcin.gacek@pcz.pl (M.G.); 4 Faculty of Mechanical Engineering and Computer Science, Częstochowa University of Technology, Dabrowskiego 69, 42-201 Częstochowa, Poland; kwiatkowski@ipp.pcz.pl (D.K.); mgucwa@spaw.pcz.pl (M.G.); 5 Synchrotron Light Research Institute, Muang, Nakhon Ratchasima 30000, Thailand; jitrin@slri.or.th

**Keywords:** geopolymer, microstructure analysis, phase analysis, electrical conductivity, sintering temperature

## Abstract

This paper clarified the microstructural element distribution and electrical conductivity changes of kaolin, fly ash, and slag geopolymer at 900 °C. The surface microstructure analysis showed the development in surface densification within the geopolymer when in contact with sintering temperature. It was found that the electrical conductivity was majorly influenced by the existence of the crystalline phase within the geopolymer sample. The highest electrical conductivity (8.3 × 10^−4^ Ωm^−1^) was delivered by slag geopolymer due to the crystalline mineral of gehlenite (3Ca_2_Al_2_SiO_7_). Using synchrotron radiation X-ray fluorescence, the high concentration Ca boundaries revealed the appearance of gehlenite crystallisation, which was believed to contribute to development of denser microstructure and electrical conductivity.

## 1. Introduction

Generally, Ordinary Portland Cement (OPC) allows sufficient thermal stability for most typical applications. However, at elevated temperatures, the properties of OPC fall off due to physical and chemical changes [1]. Several studies were conducted to identify an alternative material which possesses outstanding thermal stability and fire resistance in elevated temperature. Geopolymers are an amorphous three-dimensional (3D) aluminosilicate system, which was synthesised at ambient or slightly higher temperature by alkaline activation of suitable precursor raw material from industrial waste, fly ash [2,3,4,5,6], slag [7,8], and kaolin [9,10]. It was shown that these inorganic materials that were identified, delivered an exceptional performance at elevated temperatures.

According to Davidovits [11], the geopolymer material delivered the ceramic behaviour with the existence of crystalline phases up to 1000 °C. When heated or sintered at elevated temperatures, the transformation of the crystalline phase began to exist. Presently, Abdulkareem et al. [12] studied the fly ash geopolymer after having been heated up from ambient temperature to 800 °C without focussing on the changes in the phase composition, and element distribution occurred at elevated temperatures. Wang et al. [13] found that the structure of kaolin was majorly influenced by the calcination temperature. The change of aluminium species affected the structural changes of the geopolymer instead of the silicon atoms after having been heated at 900 °C. Meanwhile, the crystallinity behaviour and microstructural change of the slag-based geopolymer at high-temperature conditions were investigated by Rovananik et al. [14]. The calcium aluminosilicate framework filled the pores between akermanite crystals after having been heated up to 1200 °C. The dense heated geopolymer displayed a glassy phase, which is the basis of ceramic. Traditionally, ceramic vitrification is commonly initiated from 900 °C on, which was marked by the melting of several solid phases that bound the present solid particles and led to enhancing the bonding strength [15,16].

Meanwhile, according to Cui et al. [17], the electrical conductivity of geopolymer materials is intensively influenced by the microstructure appearance. The acceptable level of electrical conductivity is believed to play a major role in fast ionic conduction used for electrochemical sensors or solid-state batteries. Vladimirov et al. [18] reported that the overall electrical conductivity vitally depends on the density and nature of the grain boundaries. Understanding the microstructural and phase evolution at high sintering temperature towards electrical conductivity is strongly valuable for geopolymer and ceramic material. 

Thus, the aims of this current work were to characterise the microstructural change, crystallographic evolution, and electrical conductivity at 900 °C sintering temperatures for kaolin, slag, and fly-ash-based geopolymers. Based on these results, the correlation between crystallography and electrical conductivity was clarified. The obtained observations were correlated with the element distribution obtained using the synchrotron source of micro-X-ray fluorescence. 

## 2. Experimental Details

### Materials and Sample Preparation

The samples used in this were formed by precursor sources of kaolin (Associated Kaolin Industries Sdn Bhd, Perak, Malaysia), fly ash (Manjung Power Station, Perak, Malaysia), and slag (Ann Joo Integrated Steel Sdn Bhd, Penang, Malaysia) with the chemical composition as determined by the benchtop X-ray fluorescence spectrometer; PW4030 (Table 1). The sodium silicate (Na_2_SiO_3_) solution used was provided by South Pacific Chemical Industries Sdn. Bhd., Malaysia, consisting of SiO_2_ (30.1%), Na_2_O (9.4%), and H_2_O (60.5%) with SiO_2_/Na_2_O = 3.2. The NaOH clear solution was mixed with the sodium silicate solution and cooled down to ambient temperature one day before mixing.

The samples were designed based on three various mix geopolymer designs. Each raw aluminosilicate (kaolin, fly ash, and slag) powder, encompassing NaOH molarity, alkaline activator, solid-to-liquid ratio, and curing temperature were based on previous works and are tabulated in Table 2. The designated mixing design was based on the optimum mechanical performance [19,20,21]. The precursor material was mixed with the alkaline activator solution for 5 min. The homogenised mixture was poured into a mould. Next, after curing for 28 days, the geopolymer was crushed and sieved at 38 µm to produce a fine geopolymer powder. Two grams of each geopolymer powder were weighed and compressed via the powder metallurgy. Then, the samples were sintered at 900 °C (heating rate of 10 °C/min and soaking time for 2 h) by using a standard electrically heated furnace.

## 3. Characterisation and Analysis

The microstructure surface analysis of the unsintered and sintered mixtures was performed using the JSM-6460LA model Scanning Electron Microscope (JEOL, Peabody, MA, USA) connected with secondary electron detectors. The samples were placed onto a double-sided carbon tape. The acceleration voltage and working distance were fixed at 10 kV and 10 mm, respectively. 

The samples were examined from 400–4000 cm^−1^ (resolution of 4 cm^−1^) by using a Perkin Elmer FTIR Spectrum RX1 Spectrometer, Llantrisant, UK. The samples were prepared in powder form and then placed onto the sample slot (ATR crystal). Samples were evaluated by applying the potassium bromide (KBr) pellets methodology. The shifting of the functional group of unsintered and sintered geopolymer was recorded.

The Brunauer–Emmet–Teller (BET) surface area and pore volume were measured by the nitrogen adsorption-desorption isotherm at 77 K volume (TrisStar 3000, Micrometrics Instrument Corporation, Norcross, GA, USA). The quantity adsorbed was correlated to the total surface area and pore volume of the particles in the samples. 

The sample was fabricated in powder form for the phase analysis. The XRD analysis was performed by using an XRD-6000 Shimadzu X-ray diffractometer (Columbia City, IN, USA) (Cu Kα radiation (λ = 1.5418 Å)). The analysis was operated at 40 kV, 35 mA at 2θ ranging between 10° to 80° (scan rate of 1°/min). The XRD pattern was analysed by using X’pert High score Plus 2.0 Software by Malvern Panalytical Ltd. (Malvern, UK).

The samples were measured at ambient temperature by using four-point probe that was set up by Keithly, which was selected due to the measurement ability without contact resistance interference. The probe spacing (s) of 1 mm was used, and a current subjected to the samples was set at 0.15 s/cm and 1 A. The following equations were used to calculate the electrical conductivity of the samples.

(1) $$\rho =2\pi s\;\left(\frac{V}{I}\right)$$

(2) $$\sigma =\frac{1}{\rho }$$

where;

ρ = resistivity,

s = probe space,

*V* = voltage,

*I* = current, and

σ = conductivity.

**Figure 1:**
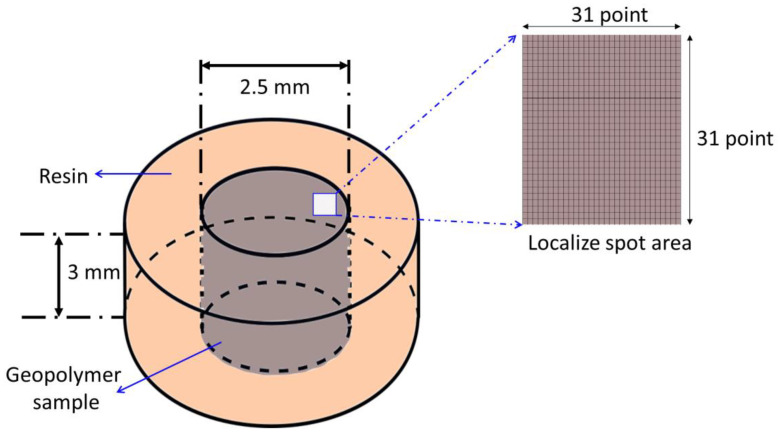
Specification of the sample for the synchrotron micro-X-ray fluorescence.

The element distributions in the sintered geopolymer were performed by using a synchrotron µ-XRF at BL6b beamline at the Synchrotron Light Research Institute (SLRI) located in Bangkok, Thailand. The detection limits at the sub-parts per million concentration level could be obtained for larger than 100 nm, and the sensitivities approached the attogram (10–18 g) level [22]. The polycapillary lens was used to initiate a micro-X-ray beam (size of 30 µm × 30 µm) on the samples and focussed the continuous synchrotron radiation. The range energy micro-X-ray beam was set (2 to 12 keV) without the monochromator feature. The experiments were conducted in a helium gas atmosphere with 30 s of exposure time at each point. The resulting images were created in bilinear interpolation and analysed using PyMca software [23]. Figure 1 depicts the sample specification and the localised scan point on the surface of the samples. 

## 4. Result and Discussion 

### 4.1. Microstructure Analysis

**Figure 2:**
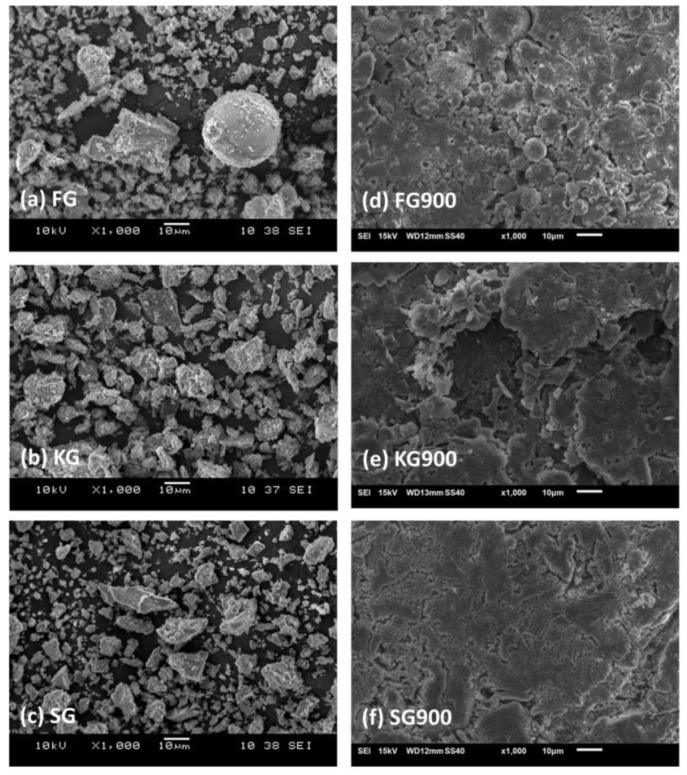
SEM micrograph of the unsintered (**a**) Fly ash geopolymer (**b**) Kaolin geopolymer (**c**) Slag geopolymer and sintered geopolymer at 900 °C (**d**) Sintered Fly ash geopolymer (**e**) Sintered Kaolin geopolymer (**f**) Sintered Slag geopolymer.

The microstructure of the fly ash, kaolin, slag geopolymers subjected to the sintering temperatures as investigated by SEM are shown in Figure 2. The unsintered fly ash geopolymer (Figure 2a) showed that there was an incomplete dissolution of the fly ash spheres, while the presence of well-defined clay platelets in the kaolin geopolymer was shown in Figure 2b. The small amount of remnant slag particles within the rough surface can be seen in Figure 2c. The remnant slag particles were enclosed within the geopolymer matrix [19]. The micrographs of the unsintered geopolymer revealed that the higher curing temperature is adequate for the development of a structurally firm geopolymer.

After sintered at 900 °C, a smooth and denser geopolymer surface could be clearly seen in the matrix, as shown in Figure 2d–f. By referring to Figure 2d, the existence of micro cracks with lesser remnant fly ash particles was observed, as indicated by the spherical-shaped particles. The kaolin geopolymer surface became increasingly glassy and smooth with the sintering temperature at 900 °C (Figure 2e). The change in the microstructure was supposedly due to the hydration of moisture and phase transformation that was discussed in the next section. Simultaneously, at the same sintering temperature of 900 °C, the slag geopolymer surface revealed lesser cracks and pore formations. The crack healing ability of the slag geopolymer caused the reduction of crack lines in conjunction with the partial melting. This result was supported by the findings of Dudek et al. [24].

### 4.2. Pore Structure Analysis

**Figure 3:**
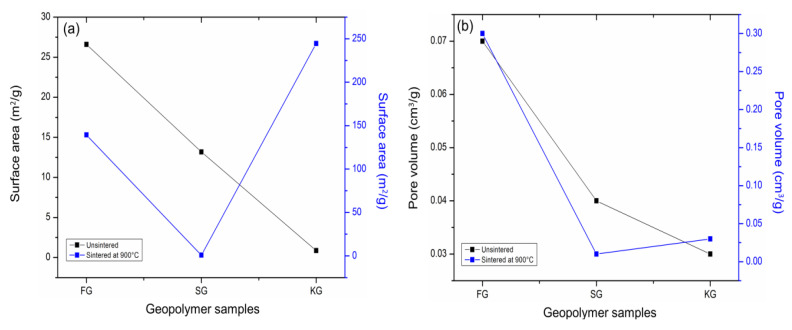
Pore structure of the geopolymer samples towards 900 °C sintering temperature; (**a**) surface area and (**b**) pore volume.

The influence of the sintering temperature (900 °C) on the pore structure of geopolymer samples was analysed by Brunauer-Emmett-Teller (BET) technology. The specific surface area and pore volume of the unsintered and sintered geopolymer are depicted in Figure 3. The Kaolin geopolymer (KG) sample obtained the lowest surface area (0.86 m^2^/g) and pore volumes (0.03 cm^3^/g), while the Fly ash geopolymer (FG) sample delivered the highest surface area (26.6 m^2^/g) and pore volume (0.07 cm^3^/g). Fly ash was the by-product of coal combustion in a thermal power plant [25], always accommodating unburned carbon, which led to a porous material that delivered a higher surface area, as shown in Figure 3. 

It can be seen in Figure 3 after having been sintered at 900 °C, Slag geopolymer (SG) showed the lowest value of surface area (0.86 m^2^/g) and pore volume (0.01 cm^3^/g) compared to KG900 and FG900. The small surface area and pore volume indicated that the SG900 is compacted and denser, thus, contributed to lower permeability due to the minimisation of porosity. This was also supported by the microstructure analysis, as shown in Figure 2c. The slag particle was believed to be consisting of mesopores-type in minor quantity, thus, obtained lower surface areas after having been introduced to the high sintering temperature.

### 4.3. Structural Spectra Analysis

**Figure 4:**
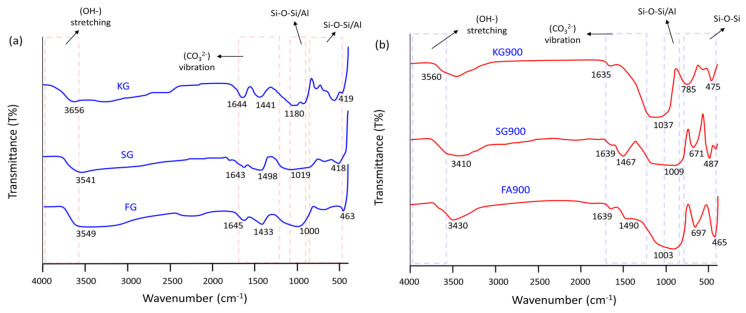
Fourier-transform infrared spectroscopy (FTIR) of the (**a**) unsintered and (**b**) sintered geopolymer at 900 °C.

The Fourier-transform infrared spectroscopy (FTIR) of the unsintered and sintered geopolymer at 900 °C is shown in Figure 4. The unsintered geopolymer presented a broad absorption band at ~1000 cm^−1^ and ~500 cm^−1^, corresponding to the huge fingerprints of the geopolymer structure, which identified as stretching vibrations of Si–O–Si/Al aluminosilicates, as depicted in Figure 4a. The weak OH^−^ stretching vibration and bending modes were determined at ~3600 cm^−1^ and ~1650 cm^−1^, respectively. These OH vibrations were found majorly because of the typical water bond absorption in the geopolymer product. Meanwhile, the narrow peaks, obtained at 1430–1500 cm^−1^, corresponded to the existence of the carbonate compound (CO)_3_^2−^. The slag geopolymer delivered the highest wavenumber at ~1500 cm^−1^, as it contained a higher level of CaO in the material, which indicated the vibration of calcite [26].

Figure 4b clearly portrays that there was a remarkable change in the absorption bands after having been sintered at 900 °C, indicating the full dehydration of geopolymer. It was evident that in the considerable broadening of the spectral region of 1000–1037 cm^−1^ in the samples sintered at 900 °C. The band shifted to a lower wavenumber; kaolin geopolymer (1037 cm^−1^), slag geopolymer (1009 cm^−1^), and fly ash geopolymer (1000 cm^−1^) due to the vibrations of Si–O–Al asymmetric telescopic and Si–O–symmetrical stretching tetrahedrally as a result of the geopolymer edifice. The existence of new bands at 700–780 cm^−1^ was attributable to the symmetrical stretching vibration of the Si–O–Si(Al) bridges corresponding to crystalline minerals such as quartz, gehlenite, and akermanite [9,27]. The appearance of these crystalline phases was further confirmed in the next phase analysis section. A typical observation was revealed for various geopolymers sintered to elevated temperatures, whereby the weak-vibration mode at 3400–3500 cm^−1^ and ~1640 cm^−1^ lowered the intensity at 900 °C, which was attributed to the OH stretching and bending vibration, suggesting the full dehydration of the geopolymer.

### 4.4. Phase Transformation

**Figure 5:**
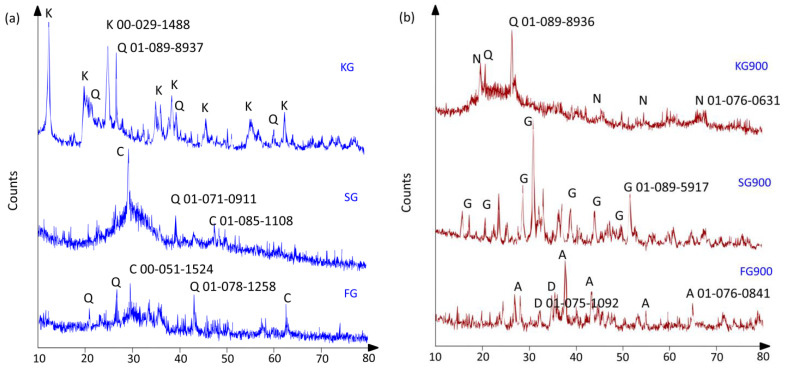
XRD diffractogram of (**a**) the unsintered geopolymer; K—kaolin (PDF No. 00-029-1488), Q—quartz (PDF No. 01-089-8937, 01-071-0911 and 01-078-1258), C—calcite (PDF No. 00-051-1524) and (**b**) the sintered geopolymer, Q—quartz (PDF No. 01-089-08936), N—nepheline (PDF No. 01-076-0631), G—gehlenite (PDF No. 01-089-5917), A—akermanite (PDF No. 01-076-0841), D—diopside (PDF No. 01-075-1092).

Figure 5a,b presents the XRD diffractograms of the unsintered and sintered geopolymers. Unsintered fly ash and slag geopolymers showed broad humps of the amorphous phase 2θ, 15–35° with several crystalline phases of quartz (Q, PDF No = 01-078-1258 and 01-071-0911) and calcite (C, PDF No = 00-051-1524 and 01-085-1108). The appearance of broad humps also corresponded with the formation of the geopolymer phase. Meanwhile, unsintered kaolin had a semi-crystalline phase diffusing halo at 20–38° 2θ, mainly of kaolinite (K, PDF No = 00-029-1428). The trace quantity of quartz (Q, PDF No = 01-089-8937) could be found in KG. The elevated temperature enhanced the propensity towards the formation of a stable crystalline phase for fly ash and slag geopolymer, portrayed in Figure 5b.

The formation of gehlenite, akermanite, and diopside could be seen in the samples sintered at 900 °C. Commonly, the evolution of the crystalline phase might act as fillers to reinforce the matrix and enhance thermal stability. The crystallisation also enhanced the denser microstructure, as exhibited in the SEM images shown in Figure 2. Based on Heah et al. [28], the crystallisation at the elevated temperature developed the mechanical properties of the geopolymer, which was believed contributed by the denser surface. 

The de-carbonation at 900 °C contributed to the decomposition of calcium hydrates and the reduction intensity of calcite. Silica, calcium oxide, and aluminium oxide were liberated from the geopolymer matrix to produce the crystalline phase of gehlenite, as shown in Equation (3) [29].

3SiO_2_·Al_2_O_3_ + 6CaO → 3Ca_2_Al_2_SiO_7_ (gehlenite) (3)

Meanwhile, akermanite was produced, as a major crystalline mineral, as a result of the reaction between calcite and free magnesium and silica oxide according to Equation (4) [30].

2CaCO_3_ + MgO + 2SiO_2_ → Ca_2_MgSi_2_O_7_ (akermanite) + CO_2_ (4)

The contradict phase analysis was obtained by KG900. Upon sintered at 900 °C, the portion of kaolinite phases transformed into semi-crystalline nepheline diffraction peaks. Based on previous studies, nepheline crystals were formed in heated-treated sodium-based geopolymers [31]. According to Sabbatini et al. [32], the existence of nepheline assisted the improvement of mechanical performance as the result of the favourable amount of silicon-rich and polymerised species. This was also corroborated with the chemical analysis of FTIR peaks, as sintered-kaolin geopolymer showed higher vibrations of Si–O–Si/Al aluminosilicates at ~1000cm^−1^ (Figure 4). 

### 4.5. Electrical Conductivity

**Figure 6:**
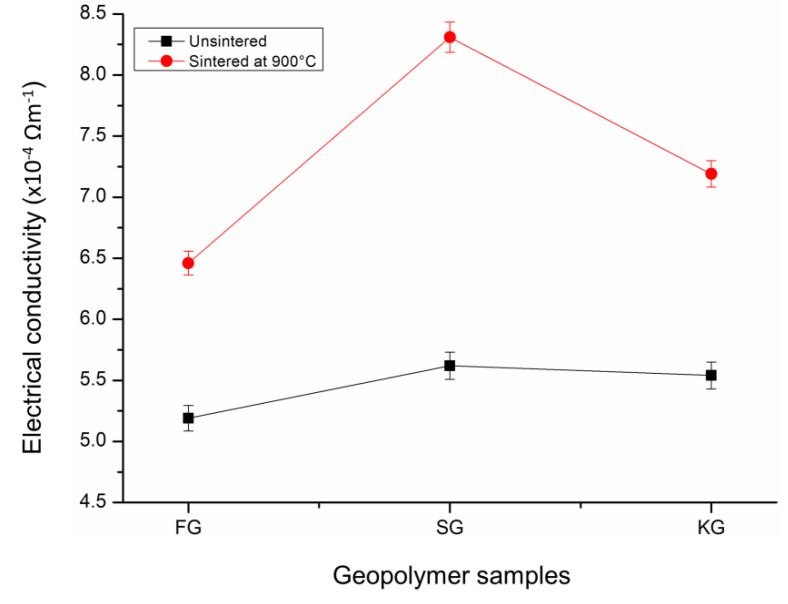
Electrical conductivity of the unsintered and sintered geopolymers at 900 °C.

The electrical conductivity was measured based on the concern of the geopolymeric matrix as potential reinforcement material in the electronic application. The result of Figure 6 shows the electrical conductivity value of the unsintered and sintered geopolymers. The highest conductivity values were depicted by SG (5.62 × 10^−4^ Ωm^−1^), while the lowest values were obtained by FG (5.19 × 10^−4^ Ωm^−1^) for the unsintered samples. The electrical conductivity in SG could be explained as a result of ion mobility (Ca^2+^, OH^−^, Na^+^) and electron transport across the percolated network of the calcium-based geopolymer. These existing ions were freely absorbed by a thin layer of the hydration product, such as calcium carbonate (CaCO_3_), as shown in Figure 4. Meanwhile, iron-rich particles in fly ash were found as the major cause for the lower electrical conductivity. The metallic iron particle was believed to exclude from the fly ash geopolymerisation. The unreacted spherical particles clearly could be visualised in Figure 2. This was also in accordance with the results published by Alida et al. [21]. 

On being sintered to 900 °C temperatures, the electrical conductivity of the geopolymers was found to increase. As it could be observed in Figure 6, SG900 exhibited a higher electrical conductivity than FG900 and KF900. For example, the electrical conductivity of SG900 was increased by 47.9%, compared to the FG900 (24.5%) and KG900 (29.8%) sintered geopolymer, respectively. The significant increase in electrical conductivity was attributed to the denser microstructure and lesser pores appearance, which connected the paths of the electron transportation and enhanced the electrical conductivity.

**Figure 7:**
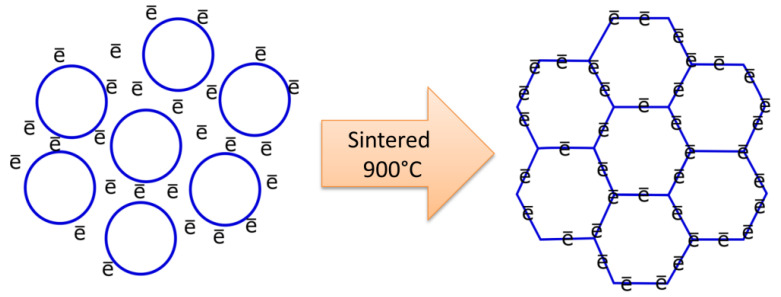
Proposed electron pathway at the crystalline grains structure at 900 °C sintering temperature.

As can be observed in Figure 7, the microstructure displayed the crystalline grains structure after the geopolymer sintered at 900 °C. The development of the ordered structure indicated the occurrence of necking grain growth, resulting in material densification [33,34]. The necking grain facilitated the additional conductive pathway on the crystalline microstructure. The structure rearrangement into the crystalline phase enhanced the alkali metal–ion transfer rate and electrical conductivity [35]. 

### 4.6. Elemental Distribution Analysis

**Figure 8:**
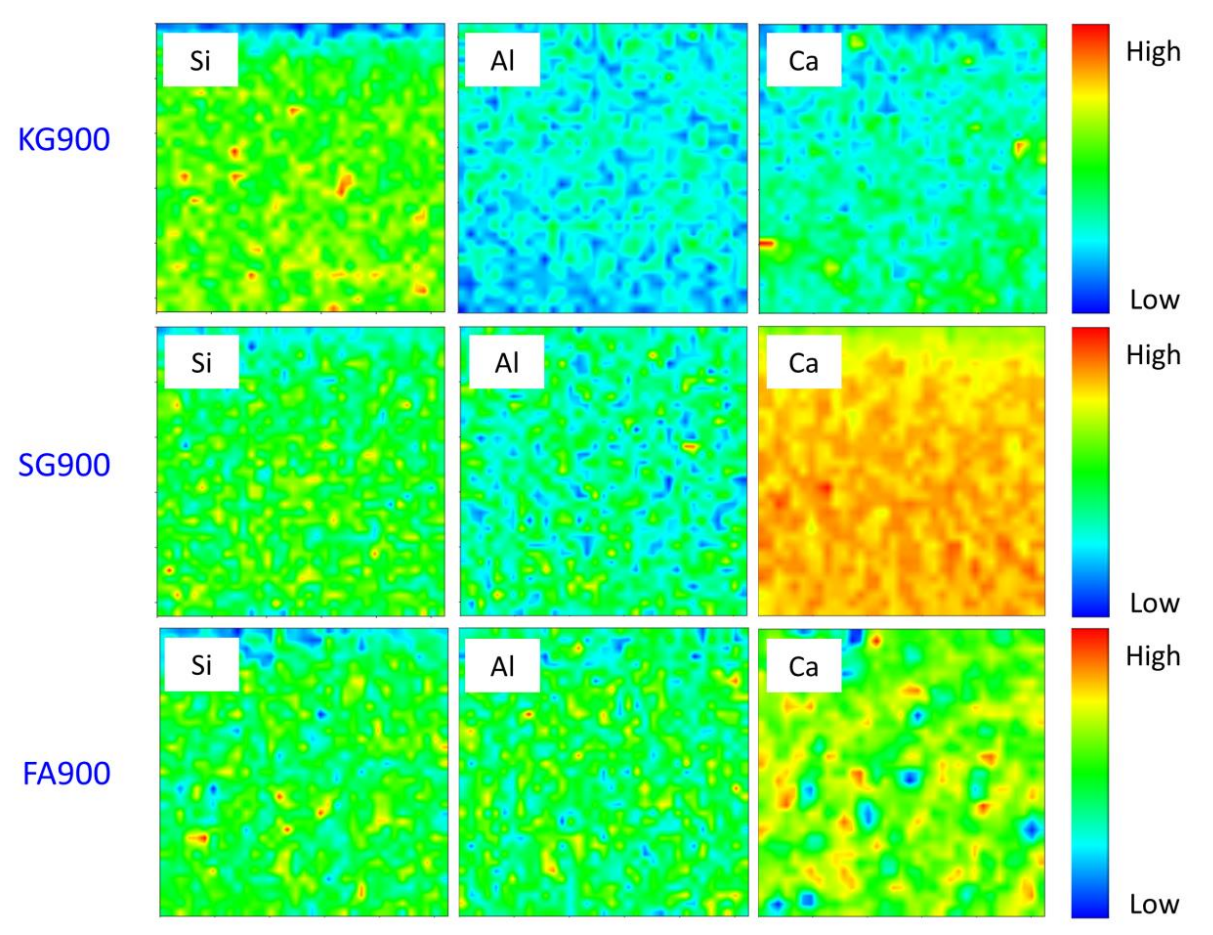
Micro-XRF elemental distribution maps of Si–Al–Ca in the sintered geopolymers at 900 °C.

The sintered geopolymer was analysed, by using the synchrotron micro-XRF analysis, in order to determine the element distribution and potential existence crystalline phase at 900 °C. Figure 8 depicts the particular area and the micro-XRF images in Si–Al–Ca of the sintered geopolymer, signifying these elements were well distributed within the samples. By using this advanced technique, the distribution of the major elements (calcium), including light elements (silicon and alumina), could be easily traced. 

By referring to Figure 8, the distribution of Si and Al was classified for the identification of the geopolymer main chain (Si–O–Al/Si). In general, the colours blue, green, and red represented low, medium, and high intensities for each distribution element at the integrated area. For KG900, the high concentrated Si region (red colour) reflected the quartz grain, while the medium concentration (green colour) of the Si element was indicated in the good homogeneity of the samples. Meanwhile, the Ca distribution was portrayed in the region of hydrated minerals of the FG900 and SG900 sintered geopolymers. The high concentrated Ca territory corresponded to the existence of gehlenite (3Ca_2_Al_2_SiO_7_) and akermanite (Ca_2_MgSi_2_O_7_). Regions with a lower concentration of Ca distribution reflected the boundaries of diopside (MgCaSi_2_O_6_), which formed a minor crystal within the crystalline geopolymer, as corroborated in Figure 5. These Ca-rich crystalline minerals were confirmed to be contributing to a denser microstructure appearance, as portrayed in Figure 2. The elevated temperature resulted in significant changes in the microstructure evolution, edging it towards the formation of a stable crystalline phase. 

## 5. Conclusions

The microstructure evolution, phase transformation, and electrical conductivity of kaolin, fly ash, and slag geopolymers at sintering temperature were analysed experimentally in this paper. The influence of sintering temperatures towards crystallisation, chemical bonding analysis, and element distribution of geopolymers were investigated. The microstructure analysis obtained the development of surface densification and lesser pores within the geopolymer matrix. The X-ray diffraction revealed that the crystallisation of gehlenite, akermanite, and nepheline was determined at 900 °C. The electrical conductivity measurement showed that the crystalline geopolymer could be proposed as a potential reinforcement material in electronic applications, especially in solder composites.

## Figures and Tables

**Table 1:** Chemical composition of kaolin, slag, and fly ash obtained by X-ray fluorescence.

Element	Mass (wt.%)		
	Fly Ash	Kaolin	Slag
SiO_2_	31.70	55.50	31.8
Al_2_O_3_	14.30	31.20	10.5
CaO	23.40	N/A	50.37
Fe_2_O_3_	23.92	4.40	0.53
K_2_O	1.54	5.41	N/A
TiO_2_	0.94	1.37	0.98
MgO	3.60	N/A	3.2
ZrO_2_	N/A	0.08	0.05
MnO_2_	N/A	0.11	0.71
LOI	1.60	1.93	1.86

**Table 2:** Mixture proportion and classification of the geopolymer.

Mix Design	Material		
	Fly Ash	Kaolin	Slag
NaOH (M)	12	8	10
Alkaline activator ratio	1.0	0.32	2.5
Solid-to-liquid ratio	2.5	1.0	3.0
Curing temperature (°C)	~25	60	~25
**Condition**	**Classification**		
Unsintered	FG	KG	SG
Sintered at 900°C	FG900	KG900	SG900

## Data Availability

The data presented in this study are available on request from the corresponding author.
